# AAPM task group report 275.S: Survey strategy and results on plan review and chart check practices in US and Canada

**DOI:** 10.1002/acm2.13952

**Published:** 2023-03-10

**Authors:** Deborah L. Schofield, Leigh Conroy, William S. Harmsen, Jennifer L. Johnson, Michelle C. Wells, Lei Dong, Luis E. Fong de los Santos

**Affiliations:** ^1^ Department of Radiation Oncology UT MD Anderson Cancer Center Houston Texas USA; ^2^ Medical Physics Princess Margaret Cancer Centre Toronto Ontario Canada; ^3^ Biostatistics, Mayo Clinic Rochester Minnesota USA; ^4^ Potentia Partners Kelburn New Zealand; ^5^ Piedmont Healthcare Atlanta Georgia USA; ^6^ Department of Radiation Oncology University of Pennsylvania Philadelphia Pennsylvania USA; ^7^ Department of Radiation Oncology Mayo Clinic Rochester Minnesota USA

**Keywords:** plan and chart check, plan review, survey results

## Abstract

**Background:**

AAPM Task Group (TG) 275 was charged with developing practical, evidence‐based recommendations for physics plan and chart review clinical processes for radiation therapy. As part of this charge, and to characterize practices and clinical processes, a survey of the medical physics community was developed and conducted. Detailed analyses and trends based on the survey that exceeded TG report length constraints are presented herein.

**Aims:**

The design, development, and detailed results of the TG‐ 275 survey as well as statistical analysis and trends are described in detail. This is complementary material to the TG 275 report.

**Methods and materials:**

The survey consisted of 100 multiple‐choice questions divided into four main sections: 1) Demographics, 2) Initial Plan Check, 3) On‐Treatment, and 4) End‐of‐Treatment Chart Check. The survey was released to all AAPM members who self‐reported working in the radiation oncology field, and it was kept open for 7 weeks. Results were summarized using descriptive statistics. To study practice differences, tests of association were performed using data grouped by four demographic questions: 1) Institution Type, 2) Average number of patients treated daily, 3) Radiation Oncology Electronic Medical Record, and 4) Perceived Culture of Safety.

**Results:**

The survey captured 1370 non‐duplicate entries from the United States and Canada. Differences across practices were grouped and presented based on Process‐Based and Check‐Specific questions. A risk‐based summary was created to show differences amongst the four demographic questions for checks associated with the highest risk failure modes identified by TG‐275.

**Conclusion:**

The TG‐275 survey captured a baseline of practices on initial plan, on‐treatment, and end‐of‐treatment checks across a wide variety of clinics and institutions. The results of test of association showed practice heterogeneities as a function of demographic characteristics. Survey data were successfully used to inform TG‐275 recommendations.

## 1 INTRODUCTION

Effective physics plan and chart review in radiation therapy is an integral component of patient safety.^1^, ^2^ Initial plan, on‐treatment (i.e., weekly), and end‐of‐treatment chart checks are critical physics activities that act as safety barriers to detect and prevent suboptimal or erroneous treatments.^3^ Following a risk‐based process proposed by the American Association of Physicists in Medicine (AAPM) Task Group (TG)‐100,^4^ AAPM TG‐275 was formed to develop national benchmarks and recommendations for the type and extent of checks to be performed for an effective physics plan and chart review in radiation therapy.

TG‐275 was also charged with conducting a survey of the medical physics community with the goal of understanding and mapping practices and clinical processes in performing plan and chart reviews. To date, only two other initiatives have provided an overview of physics plan review and chart check practices. In 2015, the results of the AAPM Safety Profile Assessment demonstrated that initial plan review and on‐treatment physics chart checks were conducted in 82 and 87 of the 114 responding institutions respectively.^5^ While this study provided some initial evidence of heterogeneity across practices with respect to plan and chart reviews, it did not provide additional information about what or how these clinical processes are performed. Also in 2015, the Medical Physics Community of Practice Chart Checking Practices Working Group (CCPWG) conducted a 36‐question survey of 15 cancer centers in Ontario, Canada.^6^, ^7^ The CCPWG survey provided information about province‐wide practices related to workload, workflow, dose verification, and patient specific Quality Assurance (QA). The CCPWG survey also investigated items checked and intra‐center variability of checks performed, troubleshooting, consultation, and documentation as part of plan review and on‐treatment chart checks. Although these two initiatives offer insight into some aspects of physics plan and chart review processes, they do not provide a full picture of the diverse practices across the medical physics community.

This work serves as a complement to the TG‐275 report providing a detailed review of the design, implementation, and results of the TG‐275 survey.^8^ Results of the survey are summarized using overall descriptive statistics. In addition, four demographic questions were selected to perform statistical tests of association to better understand differences and trends across practices.

## 2 MATERIALS AND METHODS

### 2.1 Survey scope, development, and design

The main aim of the TG‐275 survey was to gather information about how physicists perform initial plan, on‐treatment, and end‐of‐treatment chart checks for photon, electron, and proton treatment modalities across a wide variety of clinics and institutions. Brachytherapy was outside of the scope of this survey. The survey was designed and organized using information from clinical process maps developed as part of the AAPM white paper on consensus recommendations for incident learning database structures in radiation oncology.^9^ The clinical process maps provided an organized structure to capture the checks performed during the plan review and chart check processes. A comprehensive list of items to be checked was created through an iterative process of using task group members’ clinical experience combined with the premise that each step in the clinical process maps was an item to be checked. In addition to the list of check items, the survey also captured general demographics information about participant's institution and details on how plan and chart review processes are implemented at the participant's practice.

**FIGURE 1:**
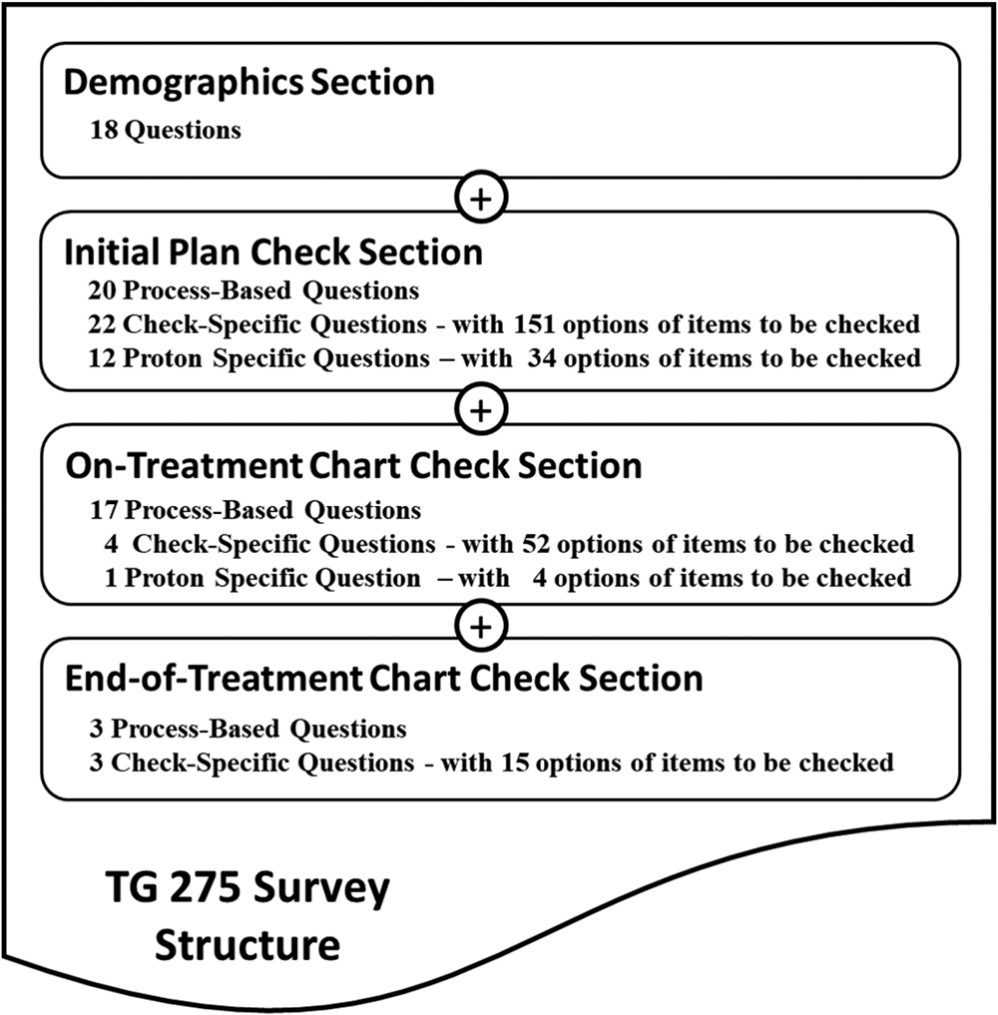
Overview of the construction of the TG‐275 survey to determine both how and what is checked during the initial plan check, on‐treatment, and end‐of‐treatment chart checks for photon, electron, and proton treatment modalities.

**FIGURE 2:**
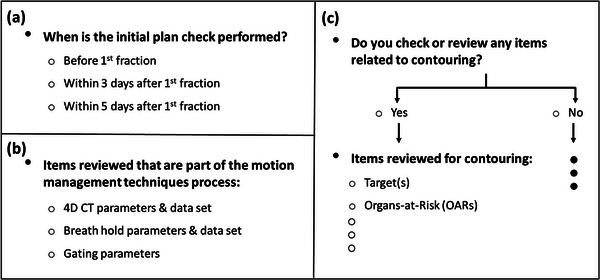
Example of Process‐Based question (a), Check‐Specific question (b), and nested question (c).

Structured data and multiple‐choice questions were used throughout the survey. Figure 1 provides an overview of the survey structure, which consisted of 100 questions divided into four main sections. The first section contained 18 demographic questions characterizing participant's facility infrastructure and general aspects of the clinical practice such as safety culture, staffing levels, patient load, and institution type (e.g., community, academic‐affiliated, etc.). The three remaining sections captured data related to the initial plan, on‐treatment, and end‐of‐treatment chart checks, respectively.  Each of these three sections contained two types of questions: Process‐Based questions, designed to determine how checks are performed, and Check‐Specific questions designed to ascertain what information is evaluated when the checks are performed. Similarly, participants were only presented with proton‐specific questions if they indicated their center provides proton therapy and that they were familiar with the proton plan review and chart check processes. Figure 2 provides an overview of the construction of nested questions and examples of the Process‐Based and Check‐Specific questions used in the survey.

Survey questions and corresponding multiple‐choice options were reviewed by TG‐275 members to ensure clarity of wording and content and to minimize survey completion time. The pre‐deployment survey completion time was estimated to range between 15 and 30 min depending on practice complexity and the number of nested questions displayed to the participant. The complete survey is provided in the Supplemental material so readers can review the questions and corresponding response options.

### 2.2 Study sample and survey participation incentives

With the support of AAPM headquarters, the survey was deployed using QuestionPro (www.questionpro.com), a web‐based survey platform. An invitation to participate was sent to all full members of the AAPM who self‐reported working, at least partially, in the radiation oncology subspecialty (approximately 4500 members). The survey was open for participation for approximately 7 weeks from 10 February to 31 March 2016.

To motivate participation and survey completion, survey respondents were enrolled in a raffle for a complementary registration to either the AAPM Annual or Spring Clinical Meeting. Additionally, respondents who completed the survey had the opportunity to fulfill Part 2 Maintenance of Certification (MOC) requirement for up to 15 h of Self‐Assessment Continuing Education (SA‐CE) and the Part 4 MOC requirement for Participatory Quality Improvement Activity as defined by the American Board of Radiology (ABR). Instructions on how to earn the credits were shared with all survey respondents who completed the survey. A Self‐Directed Educational Project template from the ABR, and an educational plan developed by TG‐275 members, were also provided. Since participants’ information was required for the raffle, the survey was not anonymous. However, the data were treated confidentially, and all responses were de‐identified for survey summary and statistical analysis.

### 2.3 Survey summary and statistical analysis

Since the TG‐275 survey used structured data and multiple‐choice questions as the main strategy to collect information, data aggregation was easily achieved.  Descriptive statistics, which included total number of responses and percentage relative to the total number of contributions to each question, were used for general demographics and plan review and chart check processes.  For the purpose of this publication and further statistical analysis, only contributions from participants in the United States and Canada were included. This data sample is consistent with the one used in the TG‐275 report to cross‐correlate the results of the failure mode and effects analysis (FMEA) risk assessment with the TG‐275 survey.

To further explore the landscape and study possible differences across clinical practices, tests of association were performed using data grouped by the following four demographic questions:

(1) ***Institution Type***: Academic and Non‐Academic clinics, where the Non‐Academic group represents participants who reported belonging to a free‐standing clinic, community hospital, or government hospital. Following other AAPM survey standards, three other options were available to choose under the institution type question: consulting groups, vendors, or other. However, participants who reported belonging to these groups were not included in the statistical analysis.
(2) ***Average number of patients treated daily***: Low (≤ 50 patients), Medium (51 to 100 patients, and High (> 100 patients) volume practices.
(3) ***Radiation Oncology Electronic Medical Record (RO‐EMR)***: ARIA and MOSAIQ.
(4) ***Perceived Culture of Safety***: Always, Usually, and ≤ Sometimes, where the “ ≤ Sometimes” group includes participants who selected Sometimes, Rarely, or Never when asked the following question: “Do you feel that there is a culture of safety in your institution where deviations and errors can be communicated amongst the groups openly and without any repercussions?”

Statistical tests of association were used to evaluate differences between the pre‐defined groups for each of the four selected demographics questions above. The statistical tests were performed for each of the 40 Process‐Based questions as well as each of the 218 checks that were common across all external beam treatment modalities. For example, statistical analysis based on Institution Type was used to assess if there were statistically significant differences in processes and items checked between participants from Academic and Non‐Academic clinics. In all cases, only univariate analysis was performed; thus, possible interdependencies between the four demographic groups were not accounted for in the determination of statistical significance (e.g., the potential interdependent relationship between Institution Type and Average number of patients treated daily was not considered). A chi‐squared test of association was performed for survey questions with discrete responses while an analysis of variance (ANOVA) test was utilized for survey questions with continuous responses. When performing the test of association on the demographic questions with three groups such as the Average Number of Patients Treated Daily (Low: < 50; Medium: 51–100; High > 100) and Perceived Culture of Safety (Always, Usually, and ≤ Sometimes), the test calculates and reports whether there is statistically significant differences across the three groups. Throughout this work, the threshold for statistical significance was *P* < 0.05.

## 3 RESULTS

### 3.1 General demographics

A total of 2030 entries were collected during the seven‐week period that the survey was open for participation. Upon review of the raw data, we found multiple entries originating from the same participant as well as non‐attributable entries with no participant demographics. As part of the data clean‐up process, all non‐attributable and duplicate entries were removed, keeping the entry with the most completed survey response. The clean data set contained 1526 non‐duplicate entries (one entry per participant), representing a 33% response rate relative to the estimated 4500 full AAPM members working in radiation oncology.  Participants from 37 countries contributed to the survey: 1310 (85.8%) from the United States, 60 (3.9%) from Canada, and 107 (7.1%) from 35 other countries. Forty‐nine participants (3.2%) did not provide a country of origin. While the clean data set included contributions from respondents in other countries, the analysis performed in this work is based only on responses from participants located in the United States and Canada (nTotal = 1370). This data sample is consistent with that used in the TG‐275 report.

Forty‐seven participants reported both working at a center with a proton facility and having experience in proton treatment delivery. Only responses from these participants contributed to the additional proton section of the survey. However, it should be noted that due to the low participation rate (47), statistical analysis of the proton‐specific checks was excluded from this work.

**FIGURE 3:**
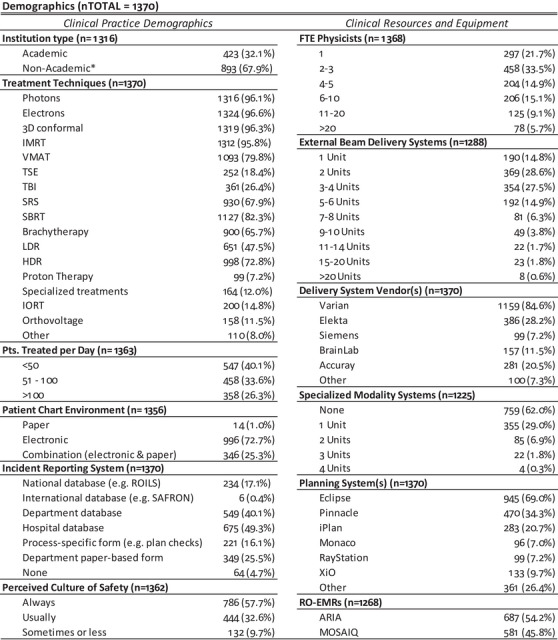
Summary of descriptive statistics for the general demographic questions. Non Academic* represents participants who reported to belong to: Free Standing Clinic, Community or Government hospital.

**FIGURE 4:**
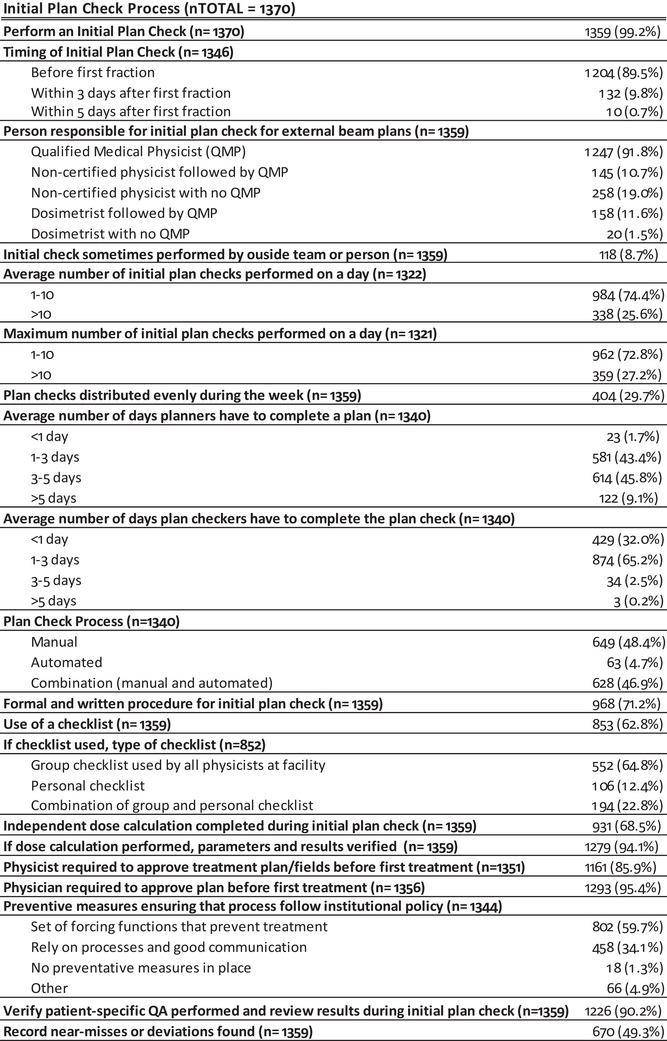
Summary of descriptive statistics for the initial plan check Process‐Based questions.

**FIGURE 5:**
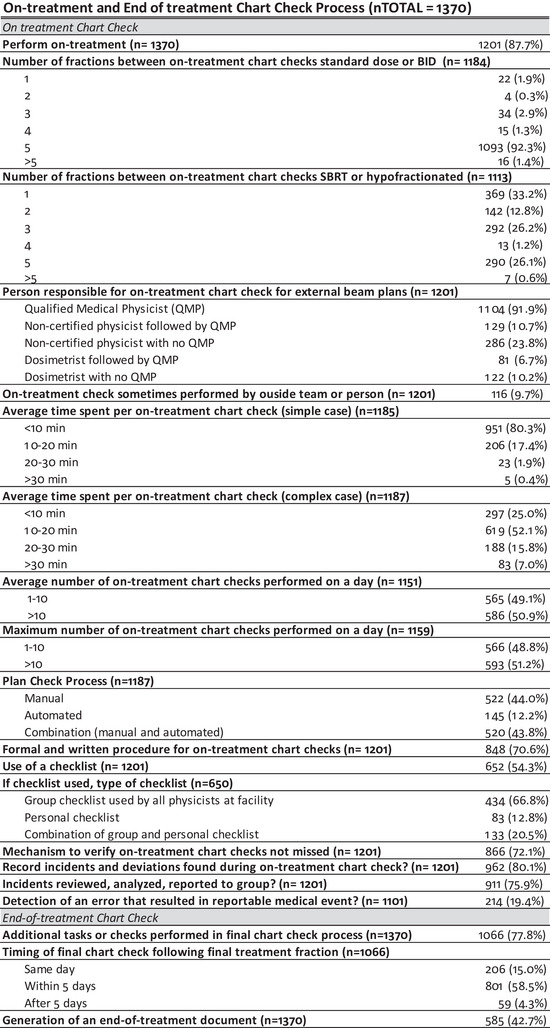
Summary of descriptive statistics for the on‐treatment and end‐of‐treatment chart checks Process‐Based questions.

Figure 3 summarizes the descriptive statistics for the general demographic questions, which included, for example, questions about the use and type of incident learning systems and participants’ perceived level of safety culture at their institution. The distribution of participants based on institution type was:  31% from academic‐affiliated hospitals, 39% from community hospitals, 19% free‐standing clinics, 7% government hospitals, 2% consulting groups, 0.1% vendors, and 1.6% other. Participants reporting belonging to the vendors group were excluded from statistical analysis because they primarily do not provide clinical services. Additionally, participants who reported belonging to consulting and other groups were excluded from the analysis to avoid possible data ambiguities given that, in the majority of instances, they could support multiple centers with different practices. A summary of the descriptive statistics for the initial plan process checks are shown in Figure 4, while the on‐treatment and end‐of‐treatment process chart checks are shown in Figure 5. To account for experimental mortality and increase accuracy, contributions to a given question were normalized using the total number of participants (n) that answered the question. For questions where participants were asked to select more than one choice, the total number of answers exceeds the number of participants and responses sum to more than 100%.

### 3.2 Differences across practices: process‐based questions

**FIGURE 6:**
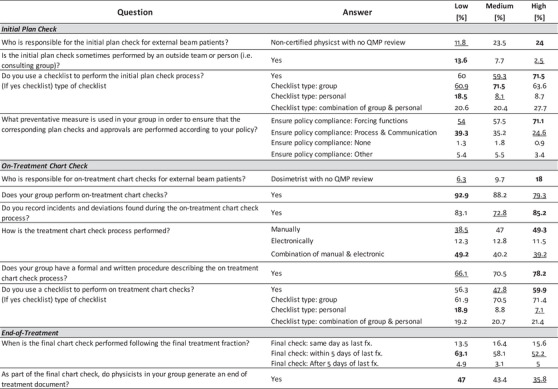
Results from test of association on Process‐Based questions using answers from the *Average Number of Patients Treated Daily* demographic question. Only showing questions where the magnitude of the intragroup difference > 10% and *P* < 0.05. (Low ≤ 50 patients, Medium between 51 and 100 patients, High > 100 patients).

**FIGURE 7:**
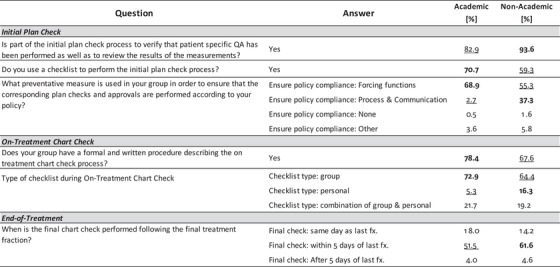
Results from test of association on Process‐Based questions using answers from the *Institution Type* demographic question. Only showing questions where the magnitude of the intragroup difference > 10% and *P* < 0.05. (Non‐Academic included answers from participants who reported belonging to a free‐standing clinic, community hospital, or government hospital.).

**FIGURE 8:**
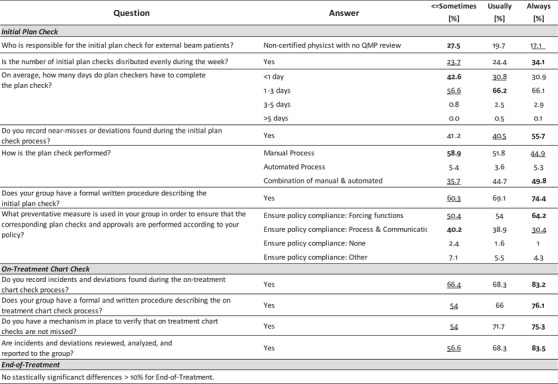
Results from test of association on Process‐Based questions using answers from the *Perceived Culture of Safety* demographic question. Only showing questions where the magnitude of the intragroup difference > 10% and *P* < 0.05. (≤ Sometimes group includes participants who selected Sometimes, Rarely, or Never).

**FIGURE 9:**
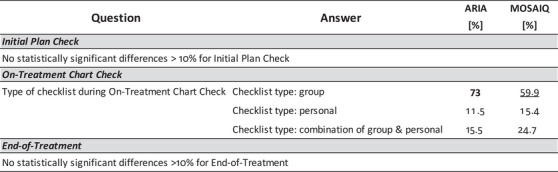
Results from test of association on Process‐Based questions using answers from the *Radiation Oncology Electronic Medical Record* demographic question. Only showing questions where the magnitude of the intragroup difference > 10% and *P* < 0.05.

Tests of association were performed for 36 of the 40 Process‐Based questions among the four demographic dimensions: Average Number of Patients Treated Daily, Institution Type, Perceived Culture of Safety, and RO‐EMR. Four questions related to individual workload (i.e., average and number of initial plans or on‐treatment checks performed per day) were excluded from this analysis. Figures 6, 7, 8, 9 summarize the differences within each demographic dimension that were found to be both statistically significant (*P* < 0.05) and where the magnitude of the intragroup difference was greater than 10%. For each question the highest percentage is **bolded** and the lowest is underlined. If one option for a given question was found to be significant and with difference > 10%, all options for that question are shown for completion, with no bolding or underlining for nonsignificant options.

Figure 6 shows process differences based on typical daily patient volume in the participant's clinic where “Low” indicates a clinic treating ≤ 50 patients, “Medium” indicates a clinic treating between 51 and 100 patients, and “High” indicates a clinic treating > 100 patients daily. Figure 7 shows process differences based on participant clinic type. “Non‐Academic” represents participants who reported belonging to a free‐standing clinic, community hospital, or a government hospital. The Perceived Culture of Safety demographic question asked participants to indicate whether deviations and errors could be communicated openly and without repercussions. Intragroup comparisons, shown in Figure 8, were made based on those who responded “Always,” “Usually,” or “≤ Sometimes” where “≤ Sometimes” grouped those who answered Sometimes, Rarely, or Never. Only a single process question had an intragroup difference > 10% that was also statistically significant (*P* < 0.05) as a function of the RO‐EMR environment, shown in Figure 9.

### 3.3 Differences across practices: summary of check‐specific questions

**FIGURE 10:**
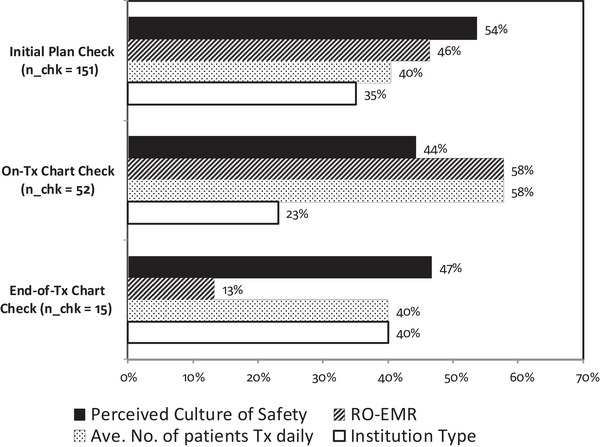
Percentage of checks shown to have statistically significant differences for each of the four selected demographic questions. n_chk = total number of checks for each of the plan review and chart check sections of the survey.

**FIGURE 11:**
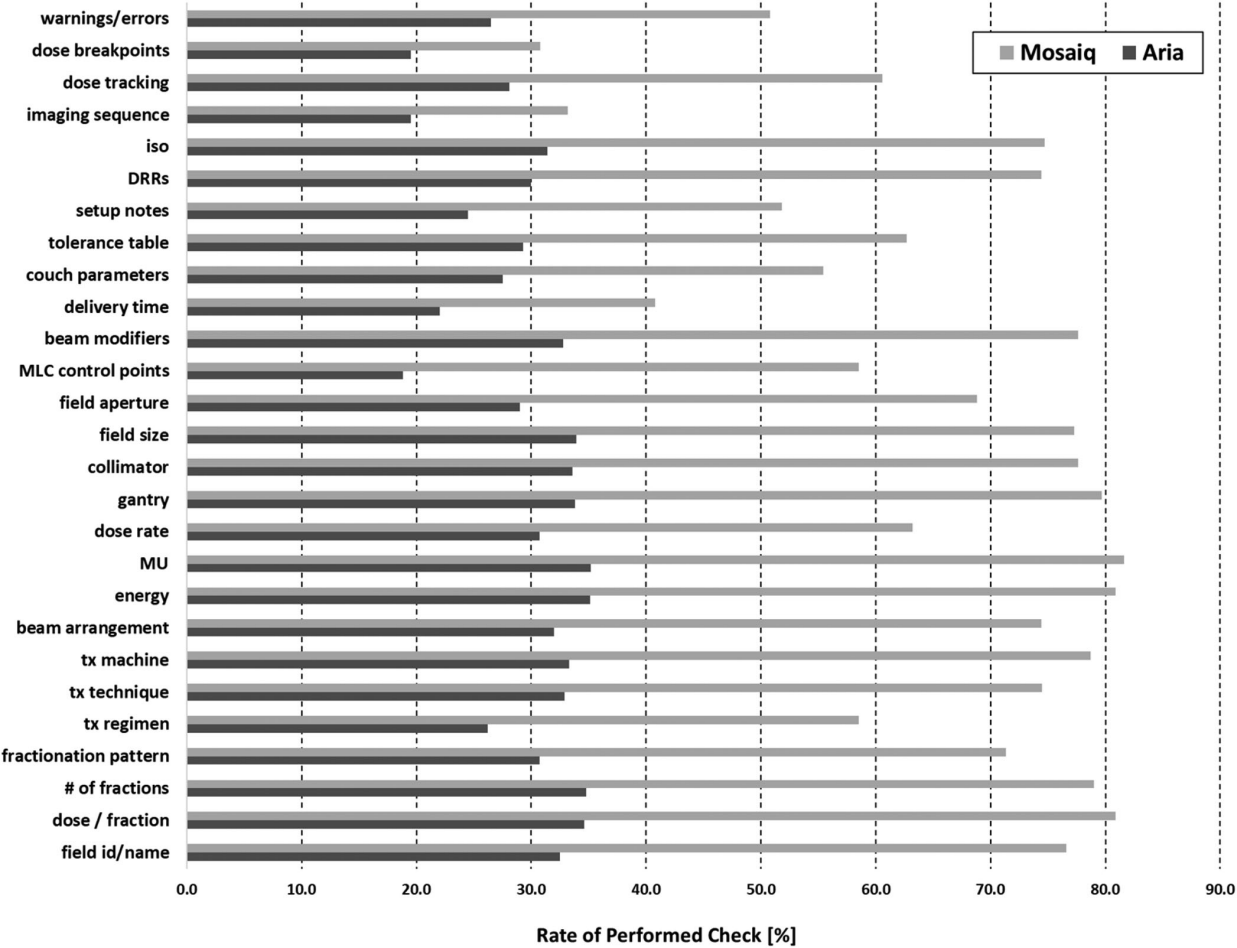
Percentage of all respondents who reported performing checks related to data transfer across third party systems as a function of RO‐EMR environment. In all cases, significantly (*P* < 0.05) more Mosaiq users perform the checks than Aria users.

In this analysis, we assessed statistically significant differences in the performance of 218 items from the initial plan check (151 items), on‐treatment chart check (52 items), and end‐of‐treatment chart check (15 items) across practices based on the four selected demographic questions: Institution Type, Average Number of Patients Treated, RO‐EMR, and Perceived Culture of Safety. For example, 66.9% of participants from Academic clinics responded “Yes” to performing a check to confirm that a plan conforms to clinical trial guidelines compared to 51.0% of participants from Non‐Academic clinics (*P* < 0.05). Figure 10 shows the percentage of checks with statistically significant differences between groups relative to the total number checks (n_chk) for each of the plan review and chart check survey sections. The Perceived Culture of Safety demographic had the largest number of statistically significant differences between groups with *P* < 0.05 for 111 checks. The RO‐EMR demographic question had 102 checks with significant differences. However, on further investigation, 27 of the checks with statistically significant differences for the RO‐EMR group were related to the verification of data transfer between third‐party systems (Figure 11). The Average Number of Patients Treated Daily and Institution Type demographics had 97 and 71 checks with statistically significant differences, respectively.

### 3.4 Differences across practices: risk‐based assessment of initial plan check

**FIGURE 12:**
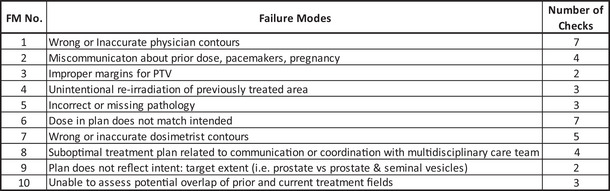
Top 10 Failure Modes and the number of checks associated with each failure mode as identified by TG‐275.

The risk‐based summary is intended to evaluate for differences amongst the four demographic questions for those checks associated with the highest risk Failure Modes (FMs) identified by TG‐275. We selected the 10 FMs with the highest Risk Priority Number (RPN) and the corresponding checks from the survey (Figure 12). A total of 40 checks were associated with the top 10 FMs. Eliminating duplicate checks that are applicable across multiple FMs resulted in a total of 28 unique checks. Twenty‐two out of the 28 unique checks met our inclusion criteria for the risk‐based summary with a statistically significant difference (*P* < 0.05) and an intra‐group difference in the performance of the checks ≥ 5% for at least one of the four demographic questions. It is important to point out that the ≥ 5% threshold is different than the > 10% threshold used in the previous section. The lower threshold of ≥ 5% was chosen to improve the sensitivity of this high‐risk based analysis.

**FIGURE 13:**
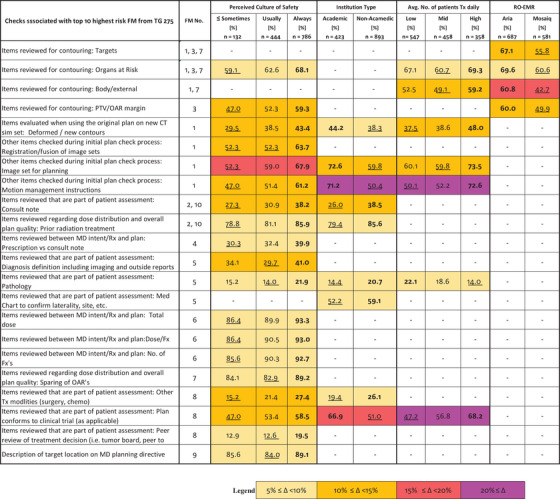
Risk‐Based Summary of initial plan review checks associated with the top 10 risk FMs where intra‐group differences in the performance of the checks ≥ 5% and the differences were statistically significant (*P* < 0.05). Checks that did not meet the inclusion criteria are left blank. Percentages listed are relative to the total number of survey contributions (n) associated with each of the demographic groups. The absolute magnitude of the difference across the demographic groups is highlighted using a color scale. The highest percentage is highlighted by bold font and the lowest percentage is underlined.

The results of the risk‐based analysis are summarized in Figure 13. Each row represents one of the 22 checks associated with a top 10 FMs that met our inclusion criteria. For each of the demographic questions, the percentage of survey participants performing the check is shown. Checks where the intra‐group differences in the performance was < 5% or the differences lacked statistical significance (*P* > 0.05) are left blank. Based on the risk‐based summary, the Perceived Culture of Safety demographic had the largest number of high‐risk checks (19 of 22) that were both statistically significant (*P* < 0.05) and had an absolute intra‐group difference ≥ 5%. The Institution Type, Average Number of Patients Treated Daily, and the RO‐EMR had 9, 7, and 4 checks, respectively, that met the inclusion criteria.

## 4 DISCUSSION

The TG‐275 survey successfully captured practices on initial plan, on‐treatment, and end‐of‐treatment checks across a wide variety of clinics and institutions. The response rate estimate of 33% is considered within the normal response rate range of widespread population surveys. External validity was evaluated by comparing the TG‐275 survey distribution to that of AAPM members who have opted to self‐report their area of practice through the organization's website.^10^ The two distributions are nearly identical with 31% and 32% of respondents working in an academic‐affiliate hospital based on the TG‐275 survey and the AAPM website respectively while 39% of participants reported working in a community practice in both data sources. Additionally, smaller clinics were well represented in the overall survey data with 70.9% of respondents reporting four or fewer treatment machines in their institution and 40.1% of survey participants indicating that their clinic treats ≤ 50 patients per day.

We selected four demographic questions on which to perform a more detailed statistical analysis. These demographic questions were selected due to their relevance to the community in general, and included: Institution type (i.e., Academic and Non‐Academic), RO‐EMR system used in the clinic (i.e., ARIA and Mosaiq), a proxy for practice workload (i.e., average number of patients treated daily), and perceived culture of safety. The analysis showed a varying number of statistically significant differences across both processes as well as initial plan, on‐treatment, and end‐of‐treatment physics check items. The demographic question with the largest number of checks with statistically significant inter‐group differences was the Perceived Culture of Safety with 111 out of 218 checks, followed by the RO‐EMR with 102, Average Number of Patients Treated Daily with 97, and Institution Type with 71 checks. Overall trends in the checks performed among the tested demographic groups were identified and are shown in Figure 10. However, this overview may not provide the full picture as some of the observed differences are due to inherent process or system‐based differences across the tested groups. For example, 27 out of the 102 checks with statistically significant differences in the RO‐EMR comparison were related to the verification of data transfer between the treatment planning system and a third‐party record and verify system.

It is also recognized that not all checks have the same level of clinical significance. The cross‐correlation exercise between the survey results and the FMEA performed by TG‐275 provides an excellent framework to classify and prioritize checks based on their risk and impact on quality and safety. Using the results of the TG‐275 cross‐correlation exercise, we were able to highlight the checks from the initial plan check process associated with the top 10 RPN failure modes. For example, while the RO‐EMR demonstrated an overall high number of checks (102 checks) with statistically significant inter‐group differences, 27 of those checks are intended to verify data transfer between the treatment planning system and a third‐party RO‐EMR. Based on the TG‐275 cross‐correlation exercise, these checks tend to have a low RPN due to the high detectability of data transfer errors. Furthermore, the risk‐based summary demonstrates that the RO‐EMR group had only four checks associated with the top 10 FMs that met out inclusion criteria (*P* < 0.05 and an intergroup difference ≥ 5%); all four checks were related to contour verification. Results from the survey seem to indicate that participants with an ARIA RO‐EMR more consistently check contouring‐related items when compared to survey participants in Mosaiq RO‐EMR environments. Based on the results of the TG‐275 survey, participants utilizing Mosaiq demonstrated working across third‐party systems at higher rates than participants utilizing Aria. As a result, an important portion of the initial plan check time in the non‐integrated environment may be used to verify data transfer potentially leaving insufficient time to perform contouring verification checks. In contrast, survey participants from integrated environments, where the RO‐EMR and the treatment planning system share the same database (e.g., ARIA‐Eclipse), may be able to dedicate more of their time to the verification of contours. Efficient access between the RO‐EMR and the treatment planning system in an integrated environment may also be a contributing factor to this observed difference.

The risk‐based summary showed that the Perceived Culture of Safety demographic group had both the largest number of statistically significant differences overall as well as checks associated with the top 10 FMs (19 out of 22 checks in Figure 13). In every instance, those who answered “Always” to whether deviations and errors could be communicated openly and without repercussions performed the checks at statistically significant higher rates than those who answered “Usually” or “≤ Sometimes.” This result seems to indicate that the ability (or inability) to openly discuss errors may be an indication of the larger, overall safety culture of a clinic. Institution Type and Average Number of Patients Treated Daily had approximately the same number of statistically significant intragroup differences associated with the top 10 FMs with 9 and 7 checks out of 22, respectively. Unlike the RO‐EMR and the Perceived Safety Culture, no common trends were identified for these two groups.

The statistical analysis and corresponding results across the 4 demographic questions (i.e., Institution Type, Average Number of Patients Treated Daily, RO‐EMR, and Perceived Culture of Safety), provides a benchmark for clinics to evaluate their own practice against other clinics as a function of demographic categories. One potential limitation of this work is that each participant's response was treated individually, and no attempt was made to aggregate data as a function of participant's institutional affiliation. This approach could potentially lead to over‐sampling bias from larger institutions. However, this data analysis approach was important to maintain participant confidentiality. Additionally, analysis of independent responses also helped ensure that individual variations in the plan review and chart check process were considered. Given that 22.8% of participants responded that plan checks were conducted using a combination of personal and institutional checklists, suggests that there can be variations between individual plan review processes within a single institution. Another limitation of the study is that a univariate analysis was utilized to provide descriptive statistics and identify trends as a function of each of the four demographic questions without evaluation of the interdependence between the variables.

The TG‐275 survey results highlight the differences in plan and chart checking practice that exist across our profession. Some of the observed variations may be due to inherent characteristics, such as differences in technology utilized in our clinics. For example, it is unclear whether the observed differences in checks of motion management instructions are due to the check not being performed or due to a lack of motion management technology. The performance of initial plan check items appears to be only moderately dependent on institutional features, such as the clinic type or daily patient volume. However, the question of whether errors and deviations can be openly discussed without fear of repercussions may be indicative of a larger safety culture issue, as 86% of high‐risk items showed significant intra‐group differences in the performance of these checks. The TG‐275 survey provided an overall picture of physics plan review and chart check practices while the result of this statistical analysis provides a more detailed picture by which an individual clinic can evaluate their own processes.

## 5 CONCLUSION

The TG‐275 survey captured a baseline of plan review and chart check practices from a large and diverse population sample across the AAPM membership. In addition to capturing data on specific checks and practices, the demographic questions allowed for further understanding of the context of those practices. In this review of United States and Canadian practices, four demographic questions were chosen for an in‐depth statistical analysis. Results of test of association showed that there is heterogeneity in the performance of these tasks as a function of demographics. The Perceived Culture of Safety had the largest number of checks with statistically significant differences including among high‐risk checks.

The underlying causes of the observed differences cannot be fully explained by the survey data. However, it does provide a rationale for future research to investigate practice variations, which may contribute to potential barriers for TG‐275 recommendations, particularly those recommendations associated with high‐risk failure modes. The TG‐275 survey also contains other demographics questions, which can form the basis of future analysis.

## AUTHOR CONTRIBUTIONS

The authors confirm contribution to the paper as follows: *Study conception and design*: Deborah L. Schofield, Leigh Conroy, Jennifer L. Johnson, Michelle C. Wells, Lei Dong, Luis E. Fong de los Santos; *Data acquisition*: Deborah L. Schofield, Jennifer L. Johnson, Michelle C. Wells, Lei Dong, Luis E. Fong de los Santos; *Analysis and interpretation of results*: Deborah L. Schofield, Leigh Conroy, William S. Harmsen, Luis E. Fong de los Santos; *Draft manuscript preparation*: Deborah L. Schofield, Leigh Conroy, William S. Harmsen, Luis E. Fong de los Santos; *Final approval of the version to be published*: Deborah L. Schofield, Leigh Conroy, William S. Harmsen, Jennifer L. Johnson, Michelle C. Wells, Lei Dong, Luis E. Fong de los Santos. All authors reviewed the results and approved the final version of the manuscript.

## CONFLICT OF INTEREST

All the authors of this manuscript do not have any conflict of interest to declare arising from the publication of this manuscript

## Supporting information

Supporting information.

## Data Availability

The data that support the findings of this study are not readily available. For questions about the data from the survey, please reach the Working Groups of Error Prevention from the American Association of Physicists in Medicine (AAPM).
