# Supplementary material for: AAPM task group report 275.S: Survey strategy and results on plan review and chart check practices in US and Canada
Source: J Appl Clin Med Phys. 2023 Mar 10;24(4):e13952. doi: 10.1002/acm2.13952 (PMC10113700; doi:10.1002/acm2.13952)
Supplement: Supplementary file 1 — Supporting information. [file ACM2-24-e13952-s001.pdf]

TG 275 SURVEY - QUESTIONS AND CORRESPONDING CHOICES

DEMOGRAPHICS SECTION

1. What treatment modalities and techniques does your practice provide (select all that apply)?

Photons
Electrons
3D conformal
IMRT
VMAT
TSE
TBI
SRS
SBRT
Brachytherapy
LDR
HDR
Proton Therapy
Specialized treatments
IORT
Orthovoltage
Other

2. Number of Photon- External beam therapy delivery systems (i.e. machines) at your site (including systems for SRS/SBRT).

2b. Select the vendors at your site for question 2.

Varian
Elekta
Siemens
BrainLab
Accuray
Other

3. Does your site provide proton therapy?

Yes
No

3a. Number of proton treatment rooms at your site:

3b. Are you familiar with the proton plan check and chart check review processes?

Yes
No

4. Number of other specialized modality delivery systems (Cyberknife, Gammaknife, Vero, etc.) in your site:

5. Select the planning system used in your institution (select all that apply):

Eclipse
Pinnacle
iPlan
Monaco
RayStation
XiO
Other

6. **Select the e-chart environment (i.e. record and verify system) (select all that apply):**

ARIA
MOSAIQ
Other

7. **Describe your institution's patient chart environment:**

Paper
Electronic
Combination (electronic & paper)

8. **Number of years that you have been practicing medical physics:**

0-2
2-5
5-10
>10

9. **Location of your practice:**

USA
Canada
Other Please specify

10. **What best describes your institution:**

Academic-affiliated hospital
Community hospital
Government hospital
Free Standing Clinic
Consulting Group
Vendor
Other please specify

11. **On average, how many patients are treated daily at your site:**

<50
51 -100
>100

12. Number of FTE physicists in your current group:

1
2-3
4-5
6-10
11-20
>20

13. Do you feel that there is a culture of safety in your institution where deviations and errors can be communicated amongst the groups openly and without any repercussions?

Always
Usually
Sometimes
Rarely
Never

14. Describe the type(s) of incident reporting system that you use in your institution (select all that apply):

National database (e.g. ROILS)
International database (e.g. SAFRON, ROSIS)
Department Database
Hospital Database
Clinical process specific form (chart checks, plan checks)
Department paper-based form
None

15. Does your group perform a formal analysis and discussion of the incidents and near misses collected by your incident reporting system?

Always
Usually
Sometimes
Rarely
Never

INITIAL PLAN CHECK SECTION

PROCESS QUESTIONS:

1. **Does your group perform an initial plan check?**

   Yes
   No

2. **When is the initial plan check performed?**

   Before 1st fraction
   Within 3 days after 1st fraction
   Within 5 days after 1st fraction

3. **Who is responsible for the initial plan check for external beam patients (select all that apply)?**

   QMP
   Non-certified physicist followed by QMP
   Non-certified physicist with no QMP
   Dosimetrist followed by QMP
   Dosimetrist with no QMP
   Other

4. **Is the initial plan check sometimes performed by an outside team or person (i.e. consulting group)?**

   Yes
   No

5. **Average number of initial plan checks performed on a day?**

6. **Maximum number of initial plan checks performed on a day?**

7. **Is the number of initial plan checks distributed evenly during the week?**

Yes
No

8. **On average, how many days do treatment planners have to complete a plan?**

<1 day
1 - 3 days
3 - 5 days
>5 days

9. **On average, how many days do plan checkers have to complete the plan check?**

<1day
1-3 days
3-5 days
>5 days

10. **How is the plan check performed?**

Manual process
Automated process
Combination (manual & automated)

11. **Does your group have a formal and written procedure describing the initial plan check?**

Yes
No

12. Do you use a checklist to perform the initial plan check process?

Yes
No

12a. Please provide the type of checklist:

Group checklist used by all physicists at your facility
Personal checklist
Combination of group and personal checklist

13. Is the independent dose calculation completed during the initial plan check process?

Yes
No

14. If the independent dose calculation has been performed prior to the initial plan check, are the parameters and results verified as part of the initial plan check process?

Yes
No

15. Is the physicist required to approve the treatment plan and/or fields prior to the first treatment?

Yes
No
NA

16. Is the physician required to approve the treatment plan prior to the first treatment?

Yes
No
NA

17. What preventative measure is used in your group in order to ensure that the corresponding plan checks and approvals are performed according to your policy?

We have a set of forcing functions that will prevent treatment
We rely on processes & good communication among the team to prevent treatment
We do not have preventive measures in place to avoid treatment
Other please specify

18. Is part of the initial plan check process to verify that patient specific QA (i.e. IMRT QA) has been performed as well as to review the results of the measurements?

Yes
No

19. Do you record near-misses or deviations found during the initial plan check process?

Yes
No

INITIAL PLAN CHECK SECTION

CHECK-SPECIFIC QUESTIONS:

The following section was intended to gather what items are checked during your Initial Plan Check Process. In order to organize the items that checked or reviewed, we classified them according to when tasks are performed in the clinical workflow. On this section you were shown tasks corresponding to the following clinical processes and asked to select all that applied.

- Patient Assessment
- Imaging for RT planning (i.e. Simulation)
- Treatment Planning

Patient Assessment

1) Items Reviewed that are part of the Patient Assessment Process:

Prescription (with respect to standard of care or institutional clinical guidelines)
Prescription approval by attending radiation oncologist
Diagnosis definition including imaging and outside records
Pathology Report
Medical Chart to confirm laterality, site, etc.
Special Considerations for radiotherapy (e.g. pacemakers, ICDs, pumps, etc.)
Previous radiotherapy treatments
Utilization of other treatment modalities (i.e. chemo, surgery)
Patient Information entered into the radiation oncology information system
Plan conforms to clinical trial (as applicable)
Patient Consent
Peer review of treatment decision (e.g. tumor board, peer-to-peer evaluation, etc.)
Consult Note
Insurance Approval
Other

Proton Specific - Patient Assessment

1) Do you evaluate the impact of implantable metallic objects?

Yes
No

Imaging for RT Planning (Simulation)

1) Items Reviewed That Are Part of the Imaging for RT planning (Simulation) Process:

Physician directive for imaging technique, setup and immobilization (this may include: contrast, scanning orientation, immobilization device, etc.)
Description of target location on physician planning directive (e.g. RUL Lung, H&N, L1-L4)
Utilization of immobilization and ancillary devices
Construction of immobilization and ancillary devices
Written or photographic documentation of patient positioning, immobilization and ancillary devices
Isocenter placement
Isocenter consistency between patient marking and setup instructions
Patient set up and positioning
Set up note
CT Scanner technique (e.g. kV, filter, etc.)
CT scan artifacts
CT scanning range (i.e. superior – inferior range includes entire target and Organs-at-Risk)
CT scan field of view and clipping of anatomy
Use of contrast and corresponding effects on HU number
Consistency between orientation of image on the CT scan and treatment plan
Transfer of image set(s) to treatment planning system
Transfer of images to archiving system
Other:

2) Items Reviewed that are part of the Motion Management Techniques Process:

4D CT parameters and data set
Breath-hold parameters and data set
Gating parameters
Other

Proton Specific - Imaging for RT planning (Simulation)

1) Please select the items that you check or review during the initial plan check process:

External metallic appendages (e.g. patient was scanned without rings or clips on ear, nose, brow, hair, tongue, etc.)
HU-proton stopping power on CT Scanner
HU- proton stopping power table on CT image recon parameters
Patient hair set up (i.e. no wet, gelled nor braided)
CT Number or relative stopping power override (e.g. high-Z material, contrast, artifacts, etc.)

Note: We are identifying nested questions by using **blue bold** font

Treatment Planning

1) Do you check or review any items related to contouring?

Yes
No

1a) Items Reviewed For Contouring:

Target(s)
Organs-at-Risk (OAR's)
Body/External contour
PTV and OAR Margin
Structures used during optimization
High-Z material, contrast, artifacts
Contours density override
Approval of contours by MD
Supporting structures (i.e. couch, immobilization and ancillary devices, etc.)
Other

2) Do you check parameters between physician intent/prescription and treatment plan?

Yes
No

2a) Items reviewed between physician intent/prescriptions and treatment plan:

Site
Laterality
Total dose
Energy
Bolus
Dose/fraction
Number of fractions
Fractionation Pattern
Additional shielding
Prescription vs consult note
Modality (e.g. electrons, photons, protons, etc.)
Technique (e.g. 3D, IMRT, VMAT, SBRT, etc.)
Regimen (e.g. BID, Quad Shot, etc.)
Other

3) Do you check or review items regarding documentation of isocenter location (shifts, multiple isocenters, etc.)?

Yes
No

3a) Items reviewed regarding documentation of isocenter location:

Additional Shifts
Multiple Isocenters
Others

4) Do you check or review optimization or calculation parameters (target and organs-at-risk objectives, algorithms, grid size, etc.)?

Yes
No

4a) Items reviewed that pertain to optimization or calculation parameters:

Target Planning Objectives
Organs-at-Risk Planning Objectives
Normalization
Calculation Algorithm
Calculation Grid Size
Density Override
Electron Density-HU Table
Other

5) Items reviewed regarding the dose distribution and overall quality of the plan:

DVH statistics
Target Coverage
Sparing of OARs
Dose Distribution
Hot Spots
Prior Radiation
Plan Sum (e.g. Original plus boost plans)
Other

Note: We are identifying nested questions by using **blue bold** font

6) Items reviewed to verify that it follows standard operating procedures of your practice or is correctly used:

Beam Arrangement
Beam Deliverability
Treatment Technique (e.g. 3D, IMRT, VMAT, SBRT, etc.)
Field ID or Name
Course and Plan ID
Setup Note
Delivery System
Beam modifiers (e.g. wedges, electron and photon blocks, tray, etc.)
MU
Energy
Dose Rate
Field Size
Field Aperture
Tolerance Table
Field Delivery Times
Bolus utilization
Collision
Setup Shifts
Treatment Couch Model
Reference Points
Dose tracking
Dose Breakpoints
Treatment plan warnings/errors
Other

7) Do you check data transfer from your planning system to a 3rd party information system (i.e. Eclipse to MOSAIQ, Pinnacle to ARIA, etc.)?

Yes
No

7a) Items reviewed to verify accurate transfer of information from one system to another:

Field ID or Name
Dose/Fraction
Number of Fractions
Fractionation Pattern
Treatment Regimen
Treatment Technique
Treatment Machine
Beam Arrangement
Energy
MU
Dose Rate
Gantry
Collimator
Field Size
Field Aperture
MLC Control Points
Beam Modifiers (e.g. wedges, electron and photon blocks, tray, etc.)
Field Delivery Times
Couch Parameters
Tolerance Table
Setup Note
DRRs
Isocenter
Imaging Sequence
Dose Tracking
Dose Breakpoints
Warnings/Errors
Other

8) Items reviewed regarding setup for image-guidance:

Matching Instructions (e.g. 2D/2D, 3D, etc.)
Reference CT
DRR association
Reference image (2D or 3D) isocenter
Imaging Technique
DRR Image Quality
Matching Structures
Imaging regimen (e.g. daily, weekly, daily followed by weekly, etc.)
Other

9) During a patient's treatment course, does your group sometimes verify that the original plan and corresponding dosimetry (i.e. DVH, target coverage, OAR sparing, etc...) is still meeting the treatment intent by using the original plan on a new simulation CT set?

Yes
No

9a) Items when your group follows the previously described process where the original plan is used on a new simulation CT set:

Old/New CT Registration
Isocenter Placement
Deformed or New Contours
DVH comparison
CTV/PTV Coverage
Organs at Risk Dose Limits
Other

9b) If the original plan on the new simulation CT set does not meet the treatment intent, does your group create a new plan in order to meet the treatment intent?

Always
Usually
Sometimes
Rarely

9c) If a new plan is created, please specify the type of check that is done on the new plan:

Full plan check
Partial plan check
No plan check

Note: We are identifying nested questions by using **blue bold** font

10) Other items that you check or review during the initial plan check process:

Registration/Fusion of image sets (CT, PET, MRI, etc.)
Image set chosen for treatment planning
Approval of physician intent/prescription
Physician designed apertures
Physics consult (e.g. evaluation of dose to pacemaker, previous treatment, etc.)
Parameters and setup for specialized devices (e.g. ExacTrac, VisionRT, RPM, etc.)
Request for in-vivo dosimetry
Motion management instructions
Treatment delivery regimen (e.g. daily, BID, regular plan follow by boost, etc.)
Verification plan for patient specific QA measurement
Transferring of treatment plan information (e.g. images, RT dose and RT structures) to archiving system
Final plan and prescription approval by physician
Second calculation check
Instruction for re-planning
Scheduling of tasks (e.g. weekly chart checks, MD image review, etc.)
Other

Proton Specific– Treatment Planning

1) Please select the items that you check or review related to contouring the body or external structure (select all that apply):

Includes everything in the beam path
Non-reproducible external objects (e.g. clothing, blankets, hair gel or wet hair, etc.)
Other

2) Items reviewed related to the beamline hardware for proton plans:

Snout Position
Snout Size (e.g. range of modulation for the specific target size)
Snout Air Gap
Lateral Expansion of the Aperture
Thickness of Collimator Aperture
Compensator (drill bit selection, manual modification of compensator, compensator smearing radius)
Settings for Range Shifter or Energy Absorber
Custom Ridge Filter
Other

3) Items reviewed as part of optimization or calculation parameters for proton plans:

Dose Calculation Volume Covers the Beam Path
HU-Proton Stopping Power Table
Dose Grid Size
Beam Calculation Model
Beam Weights
LET/Biological Dose Evaluation
Optimization Methods (e.g. SFO or MFO)
Other

4) Do you perform a plan robustness evaluation on proton plans?

Yes
No

4a) Items reviewed as part of the plan robustness evaluation for proton plans:

Individual Beam Coverage for Passive Scattering plans
Individual Beam Coverage for Scanning Beam SFO plans
Robust Evaluation for Scanning Beam plans
Other

5) Items reviewed related to the selection of a beam angle or beam direction for proton plans:

Avoiding large inhomogeneity in the path
Avoiding sharp gradient (e.g. treatment couch edge in the path)
Avoiding all beam ranging into a critical structure.
Avoiding poor modeling conditions (e.g. tangential to surface or bone, bowels, rectum)
Considering position and geometry of internal organs
Other

6) Items reviewed related to beam matching for proton plans:

Patches
Abutment
Sequence and Frequency w/ respect to total Rx
Individual dose distribution from each beam
Other

7) Do you review or check proximal, distal, and lateral beam margins for proton plans?

Yes
No

8) Do you review or check each beam range for proton plans?

Yes
No

9) Do you create and review a photon backup plan for a situation when your proton system is not available or down?

Yes
No

10) Please provide any additional items that you review or check for proton plans as part of the initial plan check process that were not covered in this section:

ON-TREATMENT CHART CHECK SECTION

PROCESS QUESTIONS:

1. **Does your group perform on-treatment chart checks?**

   Yes
   No

2. **Number of fractions treated between on treatment chart checks for standard dose fractionation or BID treatments:**

   1
   2
   3
   4
   5
   >5

3. **Number of fractions treated before the first on treatment chart check for SBRT or hypo-fractionated treatments:**

   1
   2
   3
   4
   5
   > 5

4. **Who is responsible for on-treatment chart checks for external beam patients?**

   QMP
   Non-board certified physicist with secondary check by QMP
   Non-board certified physicist with no secondary check by QMP
   Dosimetrist with secondary check by QMP
   Dosimetrist with no secondary check by QMP
   Other

5. **Is the on-treatment chart check sometimes performed by an outside team of persons (e.g. consulting group)?**

   Yes
   No

6. **Approximately, how long do you spend per on-treatment chart check (simple case)?**

<10 min
10 - 20 min
20-30 min
30 - 40 min
40 - 50 min
> 50 min

7. **Approximately, how long do you spend per on treatment chart check (complex case)?**

< 10 min
10 - 20 min
20 - 30 min
30 - 40 min
40 - 50 min
> 50 min

8. **Average number of on treatment chart check performed on a day:**

9. **Maximum number of on treatment chart checks performed on a day:**

10. **How is the treatment chart check process performed?**

Manually
Electronically
Combination (manual & electronic)

11. Does your group have a formal and written procedure describing the on treatment chart check process?

Yes
No

12. Do you use a checklist to perform on treatment chart checks?

Yes
No

12a. Please provide the type of checklist:

Group checklist used by all physicists at your facility
Personal checklist
Combination of group and personal checklist

13. Do you have a mechanism in place to verify that on treatment chart checks are not missed?

Yes
No

14. Do you record incidents and deviations found during the on-treatment chart check process?

Yes
No

15. Are those incidents and deviations reviewed, analyzed, and reported to the group?

Yes
No

16. Have you detected an incident or error that resulted in a reportable medical event?

Yes
No
Unable to answer question

ON-TREATMENT CHART CHECK SECTION

CHECK-SPECIFIC QUESTIONS:

The following section was intended to gather what items are checked during your Initial Plan Check Process. In order to organize the items that checked or reviewed, we classified them according to when tasks are performed in the clinical workflow. On this section you were shown tasks corresponding to the following clinical processes and asked to select all that applied.

- Documentation and Communication
- Plan Parameters
- Treatment Progression
- Image Guidance
- Proton Specific Items

Documentation and Communication

1) Items reviewed related to documentation and communication:

Prescription Signed
Prescription Appropriate for Treatment
Prescription Matches Plan
Case Presented at Chart Rounds
Documentation of Special Procedures (e.g. SBRT, TBI, TSE, etc.)
Weekly SSD Checks
In-Vivo Dosimetry
Modification on Prescription or Plan
IMRT QA Performed, Reviewed and Appropriate
Special instructions or needs (e.g. pacemakers, medications, chemo, blood counts, etc.)
Treatment Notes
Treatment History
Patient Alerts
Daily QA
Time-Out Performed
Other

Plan Parameters

2) Items reviewed related to plan parameters:

MU
Energy
Dose Rate
Gantry
MLC
Field Size
Collimator
Beam Modifier (e.g. wedges, electron and photon blocks, tray, etc.)
Treatment Time
Field Note
Setup Note
Plan Parameter Override
Dose Limit Override
Treatment Technique (e.g. IMRT, VMAT, 3D conformal, etc.)
Treatment Modality (e.g. photons, electrons, protons, etc.)
Couch Position Acquired at time of Verification
Couch Position (vertical/lateral/longitudinal)
Motion Management
Other

Treatment Progression

3) Items reviewed related to treatment progression:

Dose Tracking
Fraction Treated
Treatment Sessions Remaining
Partial Treatments
MD Tasks Completed
RTT Tasks Completed
Treatment Sequence (e.g. first plan follow by boost, etc.)
Treatment Regimen (e.g. BID, Quad Shot, etc.)
Changes in Prescription
Changes in Treatment Field Parameters
Revisions of Plans
Unexpected Breaks in Treatments
Manually Completed Treatments
Other

Image Guidance

4) Items reviewed related to image-guidance:

Images Approved
Shifts Applied
Treatment Ports
Registration of Images
Image Technique (i.e. CBCT, 2D kV, 2D MV)
Localization Sequence (e.g. 2D followed by 3D, 3D weekly, etc.)
Other

Proton Specific - On-Treatment Chart Check Section

4) Additional items that you check or review for proton plans as part of the on treatment chart check process:

Repeat imaging (weekly CT/MRI) performed
Adaptive RT
Pre-defined order and frequency of field specific deliveries is maintained as planned
Instruction for re-planning and tolerance levels
Other

END-OF-TREATMENT CHART CHECK SECTION

PROCESS QUESTIONS:

The End-of-Treatment Chart Check is the chart check performed upon the patient completing his/her treatment course.

1. **Does your group perform additional tasks or checks as part of the final chart check process?**

Yes
No

2. **When is the final chart check performed following the final treatment fraction?**

Same day
Within 5 days
After 5 days

3. **As part of the final chart check, do physicists in your group generate an end of treatment document?**

Yes
No

END-OF-TREATMENT CHART CHECK SECTION

CHECK-SPECIFIC QUESTIONS:

The following section is intended to gather what items are checked during your End-of-Treatment Chart Check Process. In order to organize the checks, we have classified the checks according to when the tasks are performed in the clinical workflow. Please select those items that are checked during the End-of-Treatment Chart Check Process, even if this item has already been performed or checked during other sections on the clinical workflow.

1) Items reviewed related to the documentation and communication:

End of Treatment Summary
All Documents Approved
Course Completed
All Alerts Signed Off
Treatment stopped or canceled
Data/Imaging Archived
All MD Tasks Completed
All RTT Tasks Completed
All Physics Tasks Completed
Follow up Imaging for treatment evaluation
Follow up Lab Work
Follow up Patient Management Visit
Other

2) Items reviewed related to treatment progression:

Total Dose Delivered
Total Number of Fractions Delivered
Other

3) Items reviewed related to image-guidance:

All Images Approved
Other
